# Specific Acquisition of Functional CD59 but Not CD46 or CD55 by Hepatitis C Virus

**DOI:** 10.1371/journal.pone.0045770

**Published:** 2012-09-25

**Authors:** Asim Ejaz, Eike Steinmann, Zoltán Bánki, Anggakusuma, Sana Khalid, Susanne Lengauer, Corinne Wilhelm, Heinz Zoller, Anna Schloegl, Joerg Steinmann, Elena Grabski, Michael Kleines, Thomas Pietschmann, Heribert Stoiber

**Affiliations:** 1 Institute of Virology, Innsbruck Medical University, Innsbruck, Austria; 2 Division of Experimental Virology, TWINCORE, Centre for Experimental and Clinical Infection Research, Hannover, Germany; 3 Department of Medicine, Clinical Division of Gastroenterology and Hepatology, University Hospital of Innsbruck, Innsbruck, Austria; 4 Institute of Medical Microbiology, University Hospital Essen, Essen, Germany; 5 Institute for Experimental Infection Research, TWINCORE, Centre for Experimental and Clinical Infection Research, Hannover, Germany; University of Pittsburgh, United States of America

## Abstract

Viruses of different families encode for regulators of the complement system (RCAs) or acquire such RCAs from the host to get protection against complement-mediated lysis (CML). As hepatitis C virus (HCV) shares no genetic similarity to any known RCA and is detectable at high titers in sera of infected individuals, we investigated whether HCV has adapted host-derived RCAs to resist CML. Here we report that HCV selectively incorporates CD59 while neither CD55, nor CD46 are associated with the virus. The presence of CD59 was shown by capture assays using patient- and cell culture-derived HCV isolates. Association of CD59 with HCV was further confirmed by Western blot analysis using purified viral supernatants from infected Huh 7.5 cells. HCV captured by antibodies specific for CD59 remained infectious for Huh 7.5 cells. In addition, blocking of CD59 in the presence of active complement reduced the titer of HCV most likely due to CML. HCV produced in CD59 knock-down cells were more significantly susceptible to CML compared to wild type virus, but neither replication, assembly nor infectivity of the virus seemed to be impaired in the absence of CD59. In summary our data indicate that HCV incorporates selectively CD59 in its envelope to gain resistance to CML in serum of infected individuals.

## Introduction

Worldwide about 160 million people are infected with Hepatitis C virus (HCV) [1]. HCV causes acute and chronic hepatitis which may lead to permanent liver damage and hepatocellular carcinoma. Acute HCV infection is spontaneously cleared in 50% of those patients who present with symptomatic hepatitis, whereas clearance rate is lower after asymptomatic infection [2]. Approximately 70% of patients with chronic viremia develop chronic liver disease, 10–20% of which develop liver cirrhosis [3]. To control the infection several host genetic factors were identified which are linked to the innate or the adaptive immune response such as a polymorphism near the IL28B gene, inhibitory natural killer cell receptors, or HLA class I and II alleles [4]. In the liver the innate immune response is mediated by NK and NKT cells, Kupffer cells, and infected liver cells which produce high amounts of Interferon type I and II or TNF-α [5]. Interferon α and β production is mainly induced by double stranded RNA intermediates produced during viral replication. These double stranded RNAs are recognized by TLR 3 [6], [7]. In addition NK cells may control viral spread by the release of perforin and granzymes which can kill HCV infected cells [5]. A further effector function of the innate immune system which can directly kill infected cells but also free viral particles is the complement system [8], [9], [10]. Activation of the complement can be triggered via the classical, the alternative or the MBL-pathway. All three pathways merge in the activation of C3, which is cleaved by C3 convertases into C3a and C3b. C3b becomes associated to the C3 convertase generating the C5 convertases. Then, C5 is activated by cleavage into C5a and C5b. The release of C5b initializes the terminal complement pathway resulting in formation of the membrane-attack complex (MAC) [8], [9], [10]. These late non-enzymatic process recruits sequentially C6, C7 C8 and several C9 proteins. This complex forms a pore-like structure within the envelope of the pathogen or the membrane of infected cells resulting in homeostatic breakdowns. To protect its own cells against complement-mediated lysis (CML), the host expresses several regulators of complement activation (RCA). Among them are CD46 or membrane-cofactor protein (MCP), CD55 or decay-accelerating factor (DAF), and CD59 or protectin, all of which are anchored in the membrane [8]. While MCP is inserted into the cell via a transmembrane domain, CD55 and CD59 are GPI-anchored [8]. CD55 and CD46 share similar structural motifs and consist of four short consensus repeats (SCRs) [8], [11], [12], [13]. MCP is a cofactor for the cleavage and inactivation of C3b. The role of DAF is the dissociation of the C3 and C5-convertases. In contrast, CD59 blocks integration and association of C9 to the C5–C8 complex. Thus, CD59 inhibits the formation of the lytic pore and prevents the formation of an active MAC [13]. Similar to other viral infections, HCV activates the complement system. HCV directly interacts with MBL and triggers the MBL pathway [14]. After seroconversion, HCV-specific antibodies (Abs) form immune complexes which are putative activators of the classical complement pathway. Complement activation products such as C3a or C5a and deposition of C4b and C3b are observed in HCV infected individuals and seem to be directly or indirectly involved in hepatic inflammation [15], [16]. C3a is discussed as putative prognostic marker for HCV-associated hepatocellular carcinoma [17]. All these complement-related findings clearly indicate that HCV strongly activates the complement cascades. Therefore, it is surprising that HCV is not cleared by the complement system but successfully establishes a chronic infection in most cases. As the HCV genome does not encode for any known RCAs, we tested whether the virus may acquire such RCAs from the host cell similar as described for HIV [18].

## Results

### HCV is Opsonized *in vivo*

**Figure 1:**
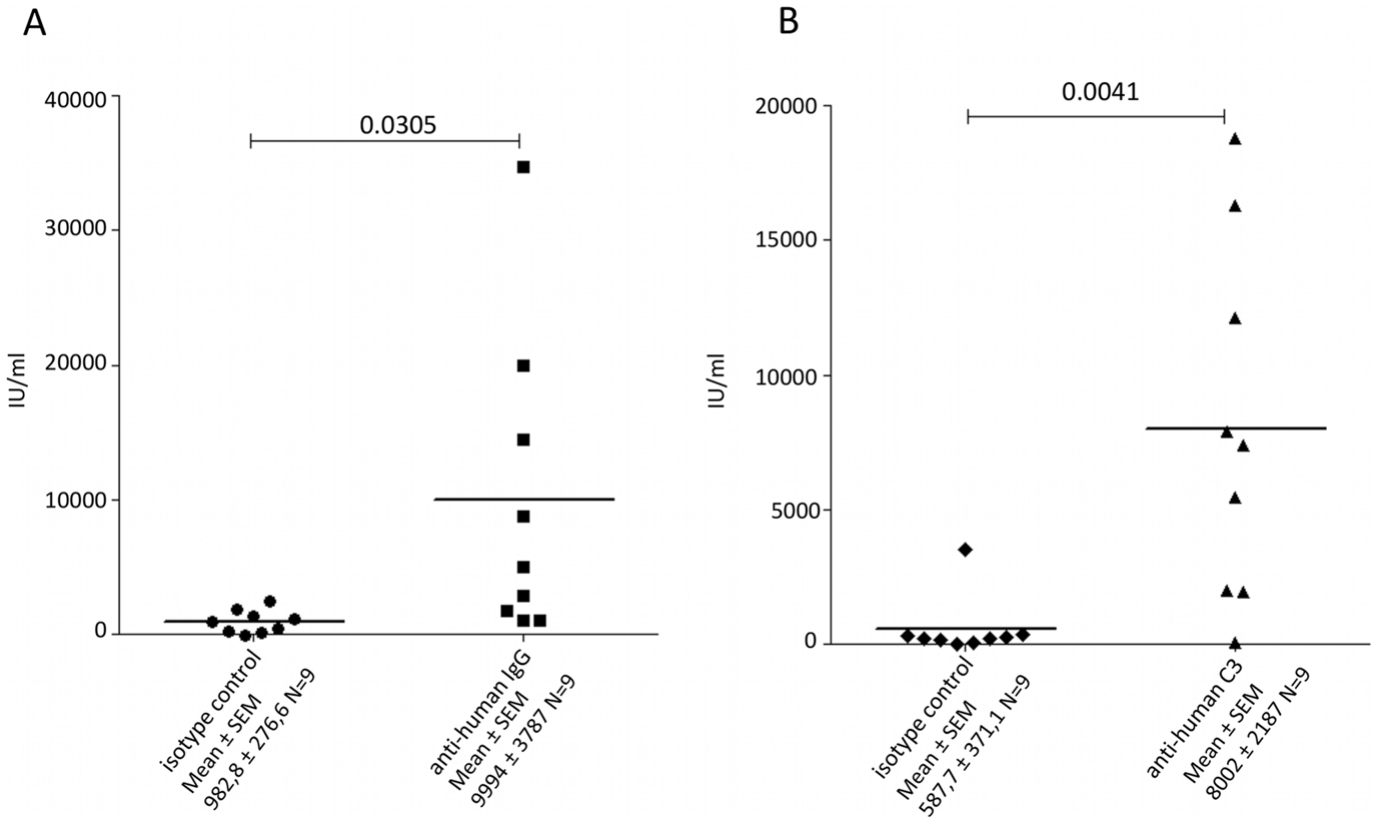
Detection of IgG and complement fragments associated with patient-derived HCV isolates. Purified HCV particles isolated from HCV infected patients were subjected to virus capture assay using 96 well plates coated with 5 µg anti-human IgG or isotype antibody *(* ***A*** *)* and anti-human C3 or isotype antibody *(* ***B*** *)*.RNA isolated from the captured wells was analyzed using Roche T*aq*Man assay. Figures represent the individual results from 9 different patient serum samples. Significance was calculated using unpaired *t*-test.

To determine if patient-derived HCV isolates are decorated with components of the complement system *in vivo*, we analyzed for the presence of C3-fragments and IgG by a modified virus capture assay (VCA) similar as described previously [19]. For this aim, purified HCV particles from positive patient samples were incubated on ELISA plates which were coated with Abs against human IgG, a mixture of C3c/C3d or isotype controls, respectively. Virus, which was opsonized by IgGs or C3-fragments, bound to the respective Ab and was thus retained in the plate. Non-captured virus was removed by a washing step. After lysis of retained HCV by detergent, the supernatant containing the released viral RNA was harvested and amplified by the Roche Taqman assay (The COBAS® AmpliPrep/COBAS® TaqMan® HCV Test). All HCV isolates tested were opsonized with both, IgG and C3-fragements independent of the HCV genotype (Fig. 1A and B, Tab. 2). This prompted us to conclude that at least a certain amount of HCV remains intact in infected individuals and is complexed with complement components.

### Detection of CD59 in the Viral Envelope

**Figure 2:**
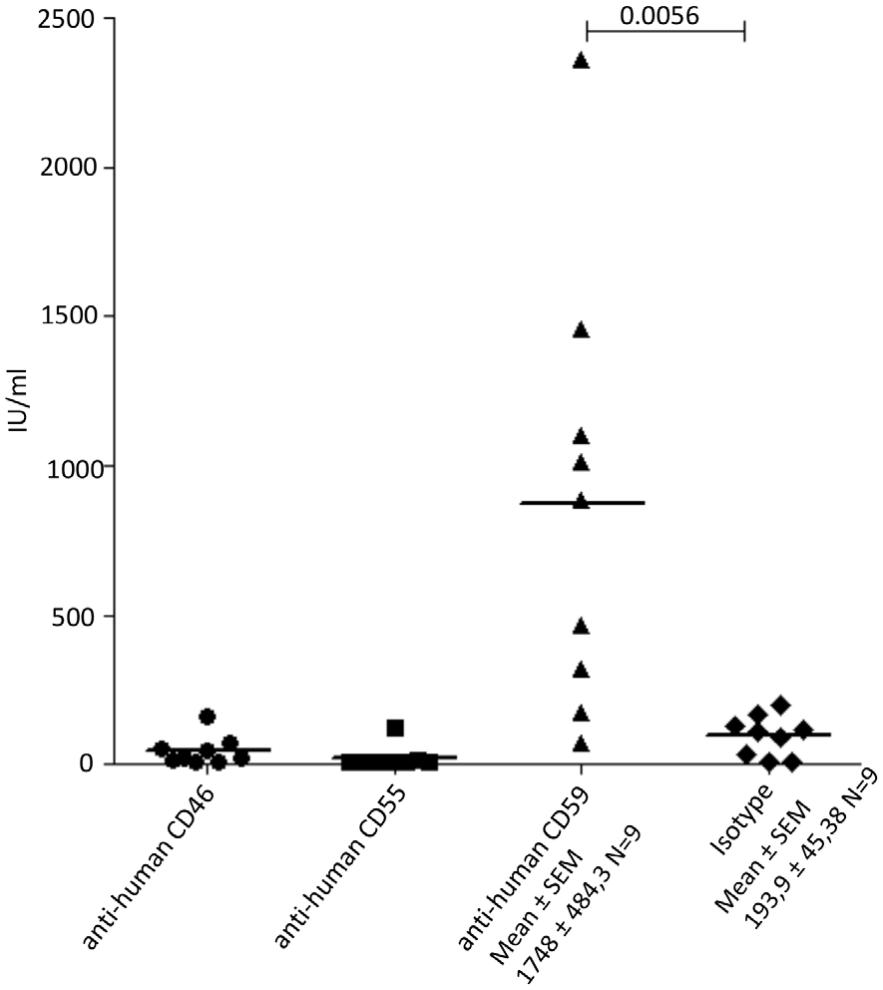
Detection of membrane bound complement regulators CD59, CD55 and CD46 associated on patient-derived HCV isolates. Purified HCV particles isolated from HCV infected patients were subjected to virus capture assay using 96 well plate coated with either 5 µg anti-human CD59, anti-human CD55 or anti-human CD46**.** RNA isolated from the captured wells was analyzed using Roche T*aq*Man assay. Figures represent the individual results from 9 different patient serum samples. Significance was calculated using unpaired *t*-test.

The stability of HCV in human serum prompted us to test for RCAs on the viral surface by a virus capture assay (VCA). In a first approach we coated plates with mAbs against the RCAs CD46, CD55 and CD59 and applied patient sera infected with different HCV genotypes (Table 1). Patient samples were incubated on ELISA plates coated with Abs against CD46, CD55, CD59 or an isotype control. Washing removed unbound HCV. HCV isolates, which had acquired any of the membrane-anchored RCAs, bound to the respective Ab and were thus retained in the plate (Fig. 2). After lysis, viral RNA was harvested and amplified again by the Roche Taqman assay (The COBAS® AmpliPrep/COBAS® *TaqMan*® *HCV* Test). HCV derived from infected individuals was positive for CD59 only, while other RCAs were not detected in this assay setup (Fig. 2). Similarly, the HCV-Jc1 supernatant harvested from infected Huh7.5 cells gave similar results (data not shown).

**Table 1:** Result of the VCAs for each patent-derived HCV isolate individually.

Patient	Geno type	IU/ml						
		Anti CD46	Anti CD55	Anti CD59	Anti C3	Isotype	Anti IgG	Isotype
1	ND^1^	41.8	15	649	12100	15	1040	484
2	1b	21	15	2030	7420	232	2890	1150
3	2	154	15	1780	16300	265	20000	15
4	1b	104	19	2200	5470	185	5070	1390
5	1b	327	247	4720	7890	341	14500	982
6	1a	98	31	936	18800	396	34790	2490
7	1a	34	15	346	1960	222	1830	300
8	3a	15	15	147	2000	15	1130	184
9	2a/2c	22	289	2920	3540	74	8790	1850

1 ND: not determined.

**Figure 3:**
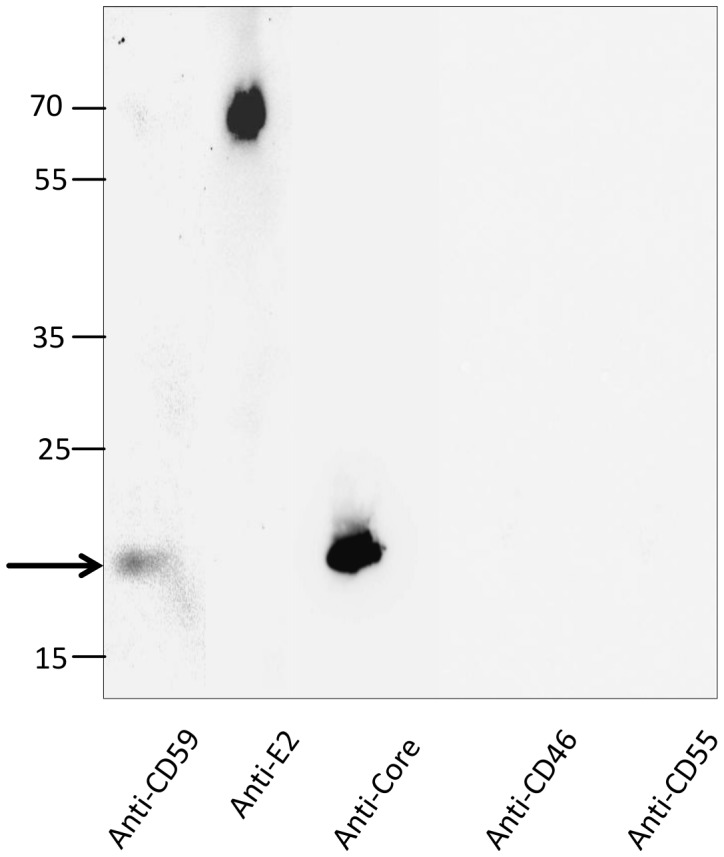
Detection of CD59 on purified HCV by Western Blot. Lysates of purified HCV were applied to Western blot analysis and developed for CD59, CD46, CD55, the viral envelope protein E2 or the HCV core protein as indicated in the figure. From the RCAs, only CD59 gave a signal at the apparent molecular weight of 20 kD, while CD55 or CD46 were not detectable.

To visualize the presence of CD59 on the HCV envelope, a Western blot was performed. Under reducing conditions, the HCV core protein showed a band at 21 kd, the viral envelope protein E2 appeared at 62 kD (Fig. 3). As expected, CD59 gave a band at 20 kD under reducing conditions (indicated by an arrow), while no signals were obtained for CD55 or CD46 (Fig. 3).

### Combined Capture-infection Assay

**Figure 4:**
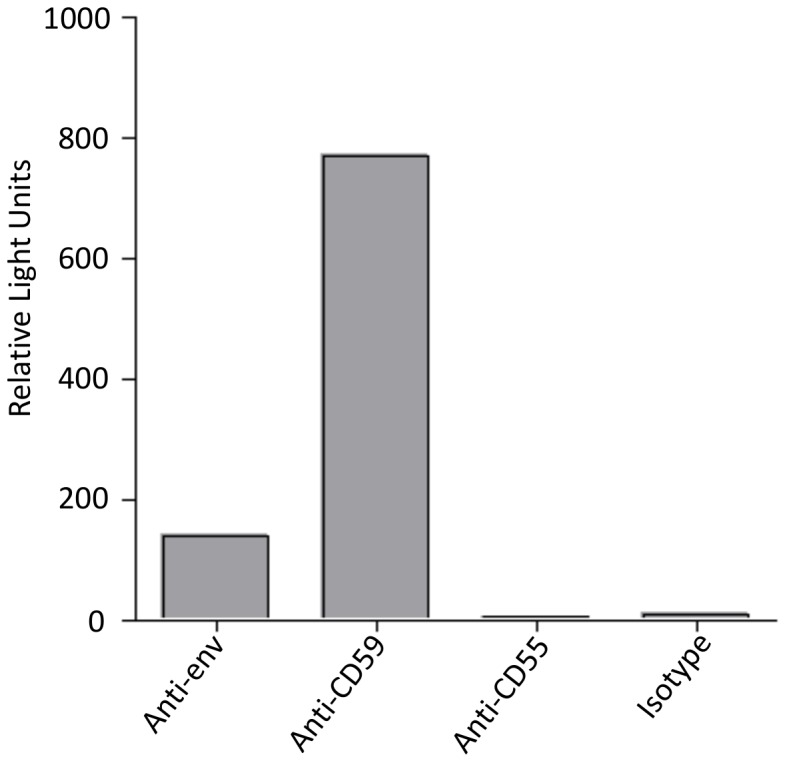
Combined virus lysis/infection assay. Luc-JC1 particles were subjected to virus capture assay using 96 well plate coated with either 5 µg anti-human CD59 or anti-human CD55. anti-HCV env (AP-33) and isotype antibodies were used as positive and negative control for the capture respectively. For detecting the captured virus, wells were overlayed with Huh 7.5 cells and infection of Huh 7.5 cells was measured by luciferase activity as relative light units. One representative experiment is shown.

To further verify the presence of CD59 on the HCV envelope we captured again the virus with mAbs against RCAs and controls, but added now HCV permissive Huh7.5 cells, which should be infected when HCV is captured and retained by the mAbs coated on the cell culture plate. Huh7.5 cells were infected by samples in which anti-env or mAb against CD59 were used. In contrast, mAbs against CD55 did not retain infectious virus. Similarly, in the control setting using isotype control antibody no infection of the cells was detectable (Fig. 4).

### Reduction of the Viral Titer by Blocking CD59

**Figure 5:**
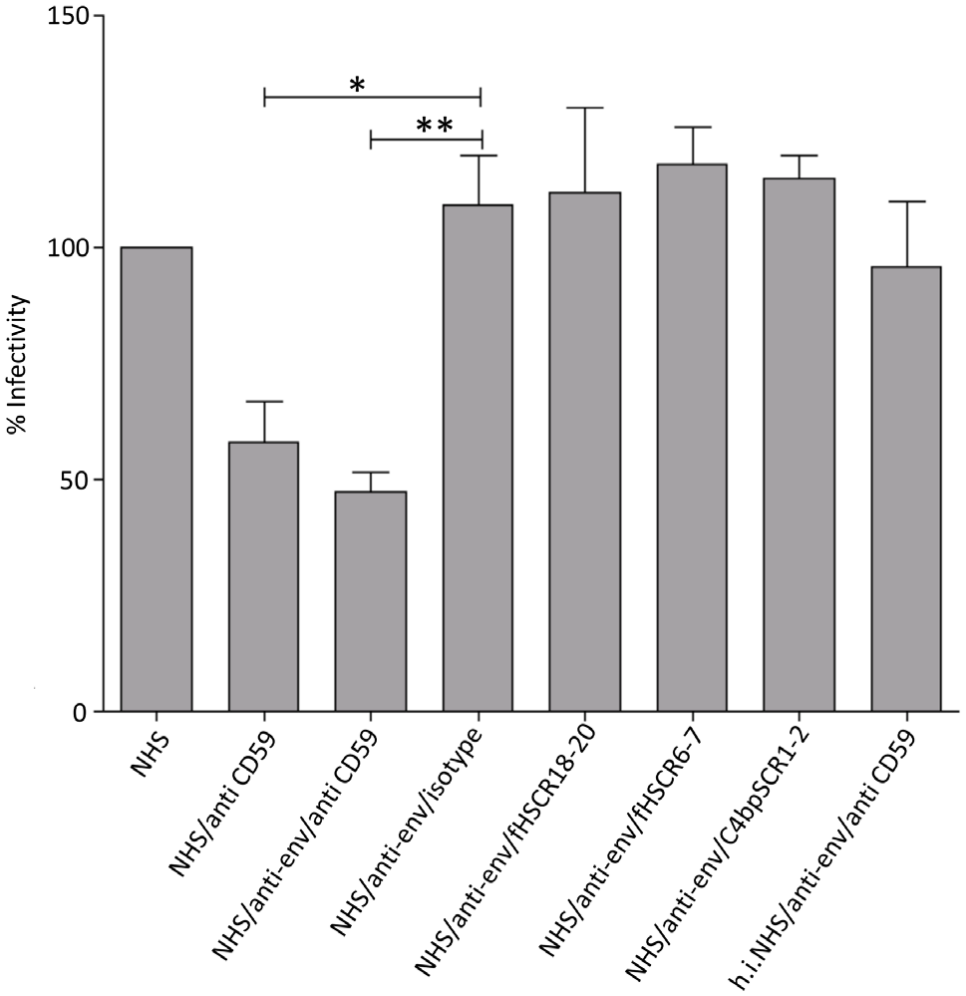
Reduction of the viral titer by blocking CD59. Cell-free culture supernatant from HCV (Luc-JC1) infected Huh7.5 cells corresponding to ∼10^4^ infectious units was incubated with anti-CD59 (MEM43), anti-env (AP-33), human Factor H SCR6–7, SCR18–20, C4 binding protein SCR 1–2 or isotype antibody either alone or in combinations, in the presence of 25% normal human serum(NHS) as source of active complement or 25% heat-inactivated normal human serum (h.i.NHS) as control. Following incubation, mixture was applied to Huh 7.5 cells. Decrease in the viral titer was estimated by measuring the luciferase activity. Infection rate with NHS alone was set at 100%. Significance was calculated using one-way ANOVA test.

The presence of CD59 on the viral envelope prompted us to test whether this RCA is still active and contributes to the protection of HCV against complement-mediated lysis. For this, HCV-Jc1 was incubated with NHS as a source of complement in the presence of anti-env and anti-CD59 Abs. After 1 hr, Huh 7.5 cells were added as a reporter cell line for HCV replication. Infection rates of viral particles treated with NHS only were set at 100%. Incubation of 15 µg/ml anti-CD59 Abs together with active serum reduced the amount of infectious particles at 42%.±8.8 (Fig. 5). The titer of HCV was further decreased by the presence of virus-specific env-Abs (clone AP-33). Since no inhibition of the infection was observed when h.i.NHS was used (Fig. 5), the moderate effect by AP-33 together with NHS was most likely due to enhanced complement activation by the antibody rather than neutralization. In line with this obersevation, AP-33 is described as non-neutralizing for the HCV isolate Luc-Jc1 [20], [21]. As close to 50% of the virus was still resistant to complement even after blocking CD59, we tried inhibiting the putative binding of the soluble RCAs fH or C4 bp. For this, fH-derived SCR6–7, SCR18–20 and SCR1–2 from C4 bp were expressed and purified from transfected *P. pastoris* and used in the lysis assay. These SCRs harbor the binding sites of fH or C4 bp to cell surfaces. Even at high molar excess (1∶50) of the SCRs compared to the concentration of the fH or C4 bp, no decrease of the infectivity of HCV was observed (Fig. 5) implying that at least theses sites in fH or C4 bp do not interact with HCV.

### Lysis and Infection Experiments Using HCV Derived from CD59-knock Down Huh7.5 Cells

**Figure 6:**
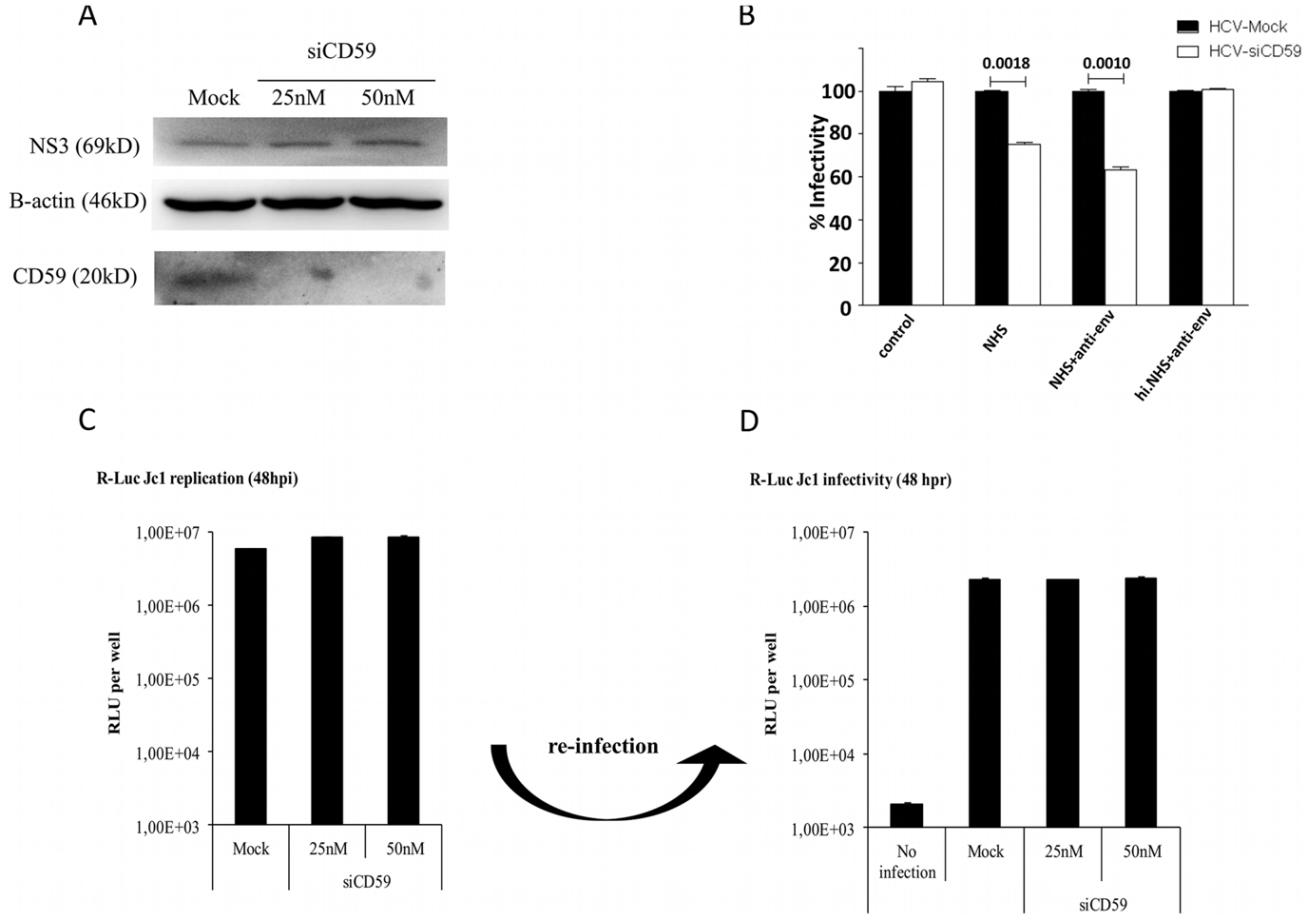
Impact of CD59 knock-down of the HCV replication cycle and virolysis. (**A**) Huh 7.5 cells were reverse-transfected with two different concentrations of siRNA against CD59 and then incubated for 72 h. Western blot analysis were performed against HCV NS3, β-actin and CD59. (**B**) The 25 nM siRNA-treated and mock treated viral supernatants were incubated with 25% normal human serum (NHS) as source of active complement or in combination with anti-env (AP33) and 25% heat-inactivated normal human serum (h.i.NHS) as control. (**C**) At 48 h post infection, the different siRNA CD59-treated cells were lysed with luciferase lysis buffer and analyzed for RNA replication efficiency by determination of luciferase acitivity. (**D**) The supernatants of transfected cells were collected and used to infect naive Huh 7.5 cells. At 48 h post infection, the cells were lysed and analysed with renilla luciferase assay for virus infectivity level.

To further confirm the contribution of CD59 in the protection of HCV against CML, Huh7.5 cells were transfected with siRNA (siCD59) to down-modulate this RCA. As determined by fluorescence analysis at a concentration of 25 nM siCD59, the signal for CD59 almost disappeared and was invisible upon treatment with a concentration of 50 nM siCD59 (Figure S1). These CD59-knock down (KD) cells were infected with HCVJc1 and the supernatant was harvested 48 h post infection. The presence of CD59 from mock-transfected and reduction/absence of CD59 of siCD59 treated as well as the infection of the cells were assessed by Western blot analysis (Fig. 6A). To induce CML, supernatants of mock- and siCD59- transfected cells were incubated with NHS as described above in the presence or absence of the HCV-specific Ab (AP33). After 1 h, the mixtures were transferred to Huh7.5 cells to allow infection of the remaining virus. Independent on the presence of AP33, the infectious titer of HCV was significantly reduced when CD59 was absent on the surface of HCV (Fig. 6B). This was in line with the results obtained with lysis/infection experiments in the presence of Abs which neutralized CD59 Abs described above (Fig. 5). To exclude that the knock-down of CD59 affects viral replication or assembly, the concentration of HCV derived from mock- or siCD59 transfected cells was determined. No reduction of the viral titers was observed, independent on the specific down-regulation of CD59 during viral replication of assembly (Fig. 6C). To assess whether the infectivity of HCV is affected in the absence of CD59, supernatants of the HCV-preparations were used to re-infect the reporter cell line Huh7.5. As shown in Fig. 6D, CD59 is dispensable for the infection process.

### Hepatocytes as a Putative Source of CD59

**Figure 7:**
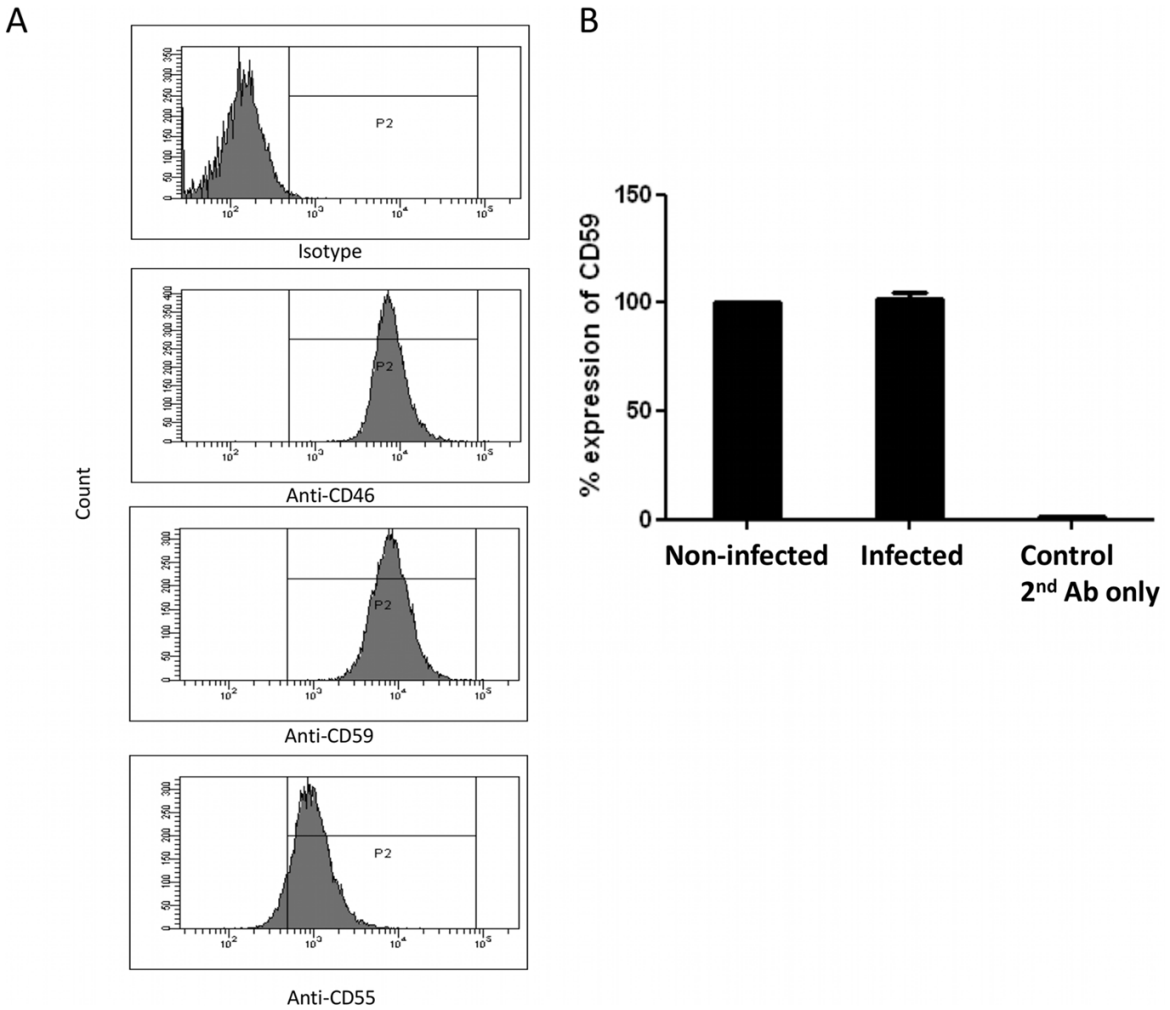
Expression of complement regulators by Huh 7.5 cells. (**A**) Surface expression analysis of membrane bound complement regulators CD46, CD55 and CD59 on non-infected Huh 7.5 cells by FACS and (**B**) comparison of CD59 expression of the cells before and after infection with HCV. Expression of CD59 on non-infected cells was set at 100%, the figure shows the mean of 3 independent experiments.

To test whether the infected cells are putative sources for the acquisition of CD59 by HCV-Jc1, we analyzed Huh7.5 cells by FACS. Staining for CD46 and CD59 revealed a prominent and comparable expression of these RCAs on the cell surface, when compared to an isotype control, while the amount of CD55 was about 10-fold lower compared to the expression of CD46 and CD59 (Fig. 7A). Expression of CD59 Huh7.5 infected with HCV-Jc1 was not up- or down-regulated indicating that HCV replication did not affect the expression profile of CD59 (Fig. 7B).

The prominent expression of the membrane-anchored RCAs prompted us to test, whether in the experimental setting used for the lysis/infection assay of Huh7.5 cells with HCV, the reporter cells are still attacked and lysed by the complement system in the presence of anti-CD59. In this case, the reduction of the viral titer might be due to the reduced amount of reporter cells in the system and not or only partially by a direct effect of CD59 blockade on HCV. To exclude this hypothesis, we repeated the lysis/infection assay analyzed now for the survival of the reporter cell line. For this, UV-inactivated HCV was incubated with freshly thawed NHS together with the antibodies against CD59, CD55 or envelope protein alone or in mixture for 1 hr at 37°C. Then, Huh7.5 cell were added and after a further incubation period of 1 h, cells were stained with propidium iodine to detect dead cells in FACS analyses. No shift of the propidium iodine signal was observed, independent on the presence of active NHS or any Ab-mixture (Figure S2). As the reporter cells were not affected, when we applied the same experimental conditions as in the neutralization/infection assay, we concluded that the reduced infectivity of HCV in the presence of NHS and anti-CD59 Abs is related to CML of the virus.

## Discussion

In the present study we provide compelling evidence that HCV acquires specifically CD59 to be protected against complement-mediated lysis upon complement activation. This complement activation was observed for all *ex vivo* HCV-isolates, independent of the genotype indicating that all HCV particles in infected individuals are opsonized. Whether the presence of IgG is a pre-requisite for C3 deposition *in vivo* is presently unclear as we had no access to HCV-isolates before seroconversion. Virus from such an early infection period would be perfect to test for C3-deposition in the absence of virus-specific IgG and to show that the HCV envelope proteins E1 and E2 can directly activate the complement. Binding of L-ficolins to the N-glycans of E1 and E2 and subsequent complement activation is reported to occur *in vitro* [15], [22]. Independent on the pathway triggered *in vivo*, the C3-deposition provides evidence of vigorous complement activation and both IgG and C3 deposition are reported to occur on hepatocytes in infected individuals. In addition, the activation maker C3a has been proposed as a biomarker for HCV-related hepatocellular carcinoma [16], [17]. Thus, HCV needs protection mechanism(s) against complement-mediated lysis. Although the HCV core protein and NS5A impair the transcription of the complement component C4 [23], the genome of HCV does not encode for any viral homologue to cellular regulators of complement activation as described for several members of the herpes- or poxvirus-family [24], [25]. Therefore, HCV may incorporate membrane anchored host-RCAs during the assembly and budding process, which are highly expressed on hepatocytes [26]. Our data provide evidence that CD59 is selectively incorporated into the membrane of HCV as, (i) the virus capture assay was selective for this RCA and does not provide any signal above background for CD46 or CD55 independent on the viral genotype; (ii) HCV retained by the virus capture assay promoted infection of susceptible Huh7.5.1 cells; (iii) blocking of CD59 in the presence of active complement reduced the viral titer most likely due to complement mediated lysis; (iv) Western blot analysis with purified HCV particles revealed the presence of CD59 on the surface of HCV; (v) HCV generated in CD59 knock down cells was significantly more susceptible to CML. The acquisition of membrane anchored CD59 is likely to differ from the uptake mechanism discussed for retroviruses, as HCV is thought to assemble and bud not from the plasma membrane [27]. It is known that upon budding into the ER lumen, viral particles are released from the cell via the secretory pathway. Hereby, the p7 and nonstructural protein 2 regulates core localization at the endoplasmic reticulum and virus assembly [28]. In line with this observation, other members of the flavivirus family built the replication complex upon a membrane scaffold rich in ER-resident protein chaperons and depends on cholesterol [29]. Interestingly, CD59 is enriched in the ER and Golgi-complex and is a marker for lipid rafts which are rich in cholesterol [30]. Recent studies have also demonstrated that virus production requires late-acting components of trans-Golgi network (TGN)-endosomal trafficking [31], [32]. Thus, CD59 is likely to co-localize with HCV proteins in the ER and golgi-complex and may be incorporated in the viral envelope at this stage of assembly. However this does not explain the selectivity for CD59 as CD55 is a marker for lipid rafts, too, and is supposed to follow similar intracellular routes and localisations [30], [33]. In contrast, CD46 is not a GPI-anchored protein and no lipid raft marker and thus may not co-localize with HCV proteins during viral assembly. CD59 is not the only cellular component acquired by HCV. The main HCV-associated lipoprotein species in serum, very low density lipoprotein (VLDL), assembles via the microsomal triglyceride transfer protein (MTP), which transfers triglycerides to nascent apolipoprotein B (ApoB) [34]. Moreover, infectious HCV virions have been shown to be associated with apolipoprotein E (ApoE), another component of VLDL [35], [36], [37]. In contrast to these lipoprotein components which are essential for the infectivity of the virus, CD59 seemed to be dispensable for viral replication, assembly or the entry process. No differences in the viral titer was observed independent on whether HCV was produced in CD59 knock down cells nor when CD59-deficient virus was used for the infection of Huh 7.5 cells. However, the presence of CD59 on both, the virus and the host cells, may reflect the necessity for protection from complement-mediated damage, as the liver is the main source of complement production and the local concentrations of complement proteins is supposed to be high [10]. Thus, CD59 might represent an important factor for HCV to survive *in vivo*.

In the presence of anti-CD59 Abs, about 50% of the virus was resistant to CML. Thus RCAs alternative to CD46, CD55 or CD59 may interact with HCV and contribute to the protection of the virus against lysis. Thus we tested whether HCV may bind soluble RCAs like fH or C4 bp, similar as described for retroviruses [38] or members of the flavivirus family [39]. To test for putative fH- or C4 bp-binding, SCR 6–7 and SCR 18–20 of fH or SCR 1–2 of C4 bp were expressed and purified. These domains were chosen as they contain binding sites to cellular surfaces, referred to as heparin binding sites [40], [41], and may thus represent the areas which are candidates as putative interaction sites of fH and/or C4 bp with HCV. Similar to CD59, the binding of the fluid phase RCAs to viral surfaces is thought to contribute to the inhibition of complement activation and thus mediate protection of viruses to CML. For Friend Virus, a member of the mouse retroviral family, SCR19–20 of fH was shown to mediate the binding of fH to the viral envelope (Huber et al, submitted). Inhibition of fH recruitment by SCR19–20 removed fH from the viral surface and allowed the complement-mediated reduction of the viral titer *in vitro* (Huber et al, submitted). As we did not observe any effects of SCR 6–7, SCR 18–20 of fH or C4 bp-derived SCR1–2 on the induction of CML, binding of these fluid phase RCAs via their heparin binding sites is not likely. Whether domains different to the SCRs mentioned above mediate a putative binding to HCV cannot be excluded presently and remains to be determined in more detail. Alternatively, RCAs different form fH or C4 bp such as clusterin or vitronection may interact with HCV and contribute to the protection of HCV against virolysis by the complement system. In this context, an interaction of clusterin with the nonstructural protein-1 of degue virus, another member of the flavivirus family was reported [42].

In summary, we report here that HCV is incorporating functionally active CD59 in its envelope as a protection mechanism against complement-mediated lysis. The acquisition of CD59 seems to be selective as neither CD55 nor CD46 were detected in cell-culture isolates or patient-derived HCV. During the preparation of the manuscript a paper by Amet et al was published in Hepatology [43] focusing on CD59 only and confirming that this RCA is acquired by HCV.

## Materials and Methods

### Cell Culture

Low passage Huh-7.5 cells (kindly provided by Charles Rice, Rockefeller University) [44] were cultured in Dulbecco’s modified Eagle medium (DMEM; Gibco) with 10% fetal bovine serum, 5% L-Glutamine, 100 µg/mL streptomycin (Invitrogen), and 100 IU/mL penicillin (Invitrogen).

### Patient Isolates

HCV infected patients gave their written consent to the study and are characterized in Table 2. The study was approved by the Ethic committee of the Medical University Innsbruck.

**Table 2:** Characterization of the patient-derived HCV isolates.

Patient	ViralLoad[IU/ml]×10^3^	IL28B	Age	Sex	Therapy	TX
1	1.014	ND^1^	ND	ND	ND	ND
2	1.425	CT	71	M	No	Yes
3	17.019	CT	63	M	No	No
4	3.890	ND	57	F	Yes	No
5	14.125	CC	58	M	No	No
6	7.624	CC	61	M	No	No
7	1.604	TT	52	F	Yes	No
8	933	CT	31	M	Yes	No
9	9.820	CC	53	F	Yes	No

1 ND: not determined.

### Virus Stocks, HCV-isolates

Sera (2 ml) from HCV positive individuals were centrifuged for 1 h (18000 rpm, 90 min, 4°C). The pellet was resuspended in RPMI1640 and further purified by a sucrose cushion. This washing step removed unbound complement proteins and IgG. The isolated viral particles were now free from soluble serum proteins, which are not directly bound to HCV, and were used for further characterization of the opsonization pattern. Construct Jc1 has been described recently [45]. Construct Luc-Jc1 encodes a chimeric HCV polyprotein which consist of codons 1–846 derived from J6/CF [46] combined with codons 847–3033 of JFH1. In this genome the HCV polyprotein-coding region is located in the second cistron and is expressed via an internal IRES element derived from the encephalomyocarditis virus. The first cistron contains the firefly luciferase reporter gene fused to the JFH1-derived 5′ NTR and coding region of the N-terminal 16 amino acids of JFH1 core [47].

### Antibodies and Normal Human Serum

Antibodies against CD55 (HD1A) and CD59 (MEM43) antibodies were a kind gift of Dr C. Harris (Cardiff University School of Medicine). Anti-HCVenv (AP-33) was a kind gift from Genentech. Mouse anti-human CD46 (clone E4.3), anti-human C3c, iC3d, were purchased from BD. Anti-human IgG, anti mouse IgG-FITC were from Dako. Normal human serum (NHS) of 6 health donors was pooled and used as a source of complement. Samples were aliquoted, stored at −80°C and thawed only once for the experiments. Some aliquots were heat inactivated (h.i.NHS; 56°C, 30 min) and served as controls.

### Expression and Purification of fH- and C4 bp-derived SCRs

The genes coding for the human factor H SCR 6–7, SCR 18–20 and human C4BP SCR 1–2 were amplified from respective cDNA using primers, designed to incorporate the restriction sites for cloning into expression vectors pPICZα A. The recombinant proteins were expressed in *Pichia pastoris* strain X-33 and purified using HisTrap HP column (GE) according to the recommendation of the manufacturer.

### Virus Capture Assay

Virus capture assay (VCA) was performed as described for the characterization of the HIV-1 envelope [19]. Briefly, a 96 well plate was coated with 100 µl per well rabbit anti-mouse (Dako), diluted 1∶180 in coating buffer (0.1 M NaCO_3_, pH 9.6) overnight at 4°C. The wells were washed 3 times with 3% skim milk powder (SMP) in phosphate buffered saline (PBS), 100 µl per well. Coated wells were incubated with 5 µg of capturing antibody either a of anti-C3c/C3d, anti-IgG, anti-CD46, anti- CD55, anti-CD59, anti-envelope protein or the isotype control Abs in 100 µl of 1% SMP/PBS for 2 hours at room. Wells were washed 3 times with PBS followed by incubation with 50 µl of either JC-1 virus culture supernatant or patient serum at 4°C overnight for the detection of RCAs on the viral envelope. To characterize the opsonization pattern, purified HCV particles isolated from HCV-infected individuals were used. Unbound virus was removed by gently washing the wells 5 times with PBS. RNA of the cell culture derived virus (HCV-Jc1) was harvested using virus RNA isolation kit (Qiagen) and amplified by real time PCR as described below. The RNA of VCAs with patient serum and patient derived-isolates was quantified using Roche T*aq*man assay (The COBAS® Ampliprep/COBAS®T*aq*Man HCV Test) according to the protocol of the manufacturer. The detection limit of the assay was 10 IU/ml corresponding to 20 copies/ml.

### Real Time PCR

RNA from the capture assay of Jc1 was tested in triplicate using forward primer S-146, 5′-TCT GCG GAA CCG GTG AGT A-3′, reverse primer A-219, 5′-GGG CAT AGA GTG GGT TTA TCC A-3′ and Probe A-195, 5′-6-Carboxy-Fluorescine- AAA GGA CCC AGT CTT CCC GGC AAT T- Tetra-Chloro-6-Carboxy-Fluorescine-3′. Reaction was performed using I-script RT PCR kit using 40 µl reaction mixture volume. The reaction was run on an iCycler iQ™ (Bio-Rad) using following settings; 10 min 50°C, 5 min 95°C, then cycled 50 times at 95°C for 15 sec, 55°C for 30 sec and 72°C for 15 sec. The data were analyzed using iCycler iQ™ data analyses module.

### Western Blot

Cell-free supernatant from Jc1 infected Huh7.5 cells was purified using 20% sucrose cushion ultracentrifugation. 1 ml of the purified virus fraction (10^8^ copies/ml) was pelleted by ultracentrifugation at 18000 rpm, 4°C for 90 min. Virus pellet was resuspended in 50 µl 1% Triton X-100 and boiled for 5 min with 5 × reducing loading buffer. Proteins were analyzed using 12% SDS-PAGE under reducing conditions followed by Western blotting. After blocking, membranes were incubated overnight at 4°C with 1 µg/ml anti-CD59, anti-CD46, anti-CD55, anti-E2 (AP-33) and anti-core (C7–50) Abs. HRP-conjugated anti-mouse IgG-Abs were used as a secondary Ab. The blots were developed using the ECL detection system (GE heathcare).

### Combined Virus Capture/infection Assay

To test whether the captured HCV Jc1 isolate retained its infectivity, a VCA was performed as described above. However instead of lysing HCV by detergents and isolating the RNA, the wells were washed vigorously with DMEM medium and incubated with Huh 7.5 cells. Cells were further cultured for 3 days and then subjected to luciferase detection assay. For assaying the luciferase activity, cells were washed once with PBS, lysed directly on the plate with 1 ml of ice-cold lysis buffer (0.1% Triton X-100, 25 mM glycylglycine, 15 mM MgSO_4_, 4 mM EGTA and 1 mM DTT, pH 7.8), and frozen. After thawing, lysates were resuspended by pipetting. For each well, 100 µl lysate was mixed with 360 µl assay buffer (25 mM glycylglycine, 15 mM MgSO_4_, 4 mM EGTA, 1 mM DTT, 2 mM ATP and 15 mM K_2_PO_4_, pH 7.8) and, after addition of 200 µl of a luciferin solution (200 µM luciferin, 25 mM glycylglycine, pH 8.0), measured for 20 s in a luminometer (Lumat LB9507; Berthold, Freiburg, Germany).

### Combined Virus Lysis/infection Assay

To elucidate whether CD59 on the viral surface has retained its functional activity and contributes to the protection of HCV against lytic attacks of the complement system, 100 µl of Jc-1 culture supernatant corresponding to ∼10^4^ infectious units were mixed with anti-CD59, anti envelope protein, isotype antibodies (15 µg/ml), human Factor H SCR18–20, 6–7, C4 binding protein SCR 1–2 (30 µg/ml) in combination or separately according to the experimental setup. Followed by 10 min incubation at room temperature 25% freshly thawed NHS was added to make the final reaction volume to 200 µl using KBR buffer (virion serion). 25% h.i.NHS was used as control. The mixture was gently shaken for 1 hr at 37°C at with a thermal block followed by the addition of 100 µl of the reaction mixture to seeded Huh7.5 cells. Cells were further cultured for 3 days and then subjected to luciferase detection assay as described above.

### Virus Lysis/infection Assay on Particles Produced from hCD59 Knock-down (KD) Cells

The hCD59 siRNA used in this assay was purchased from Dharmacon (M-004537-01). Huh 7.5 cells (2×10^5^) were reverse-transfected in 6-well plate using Lipofectamine RNAiMax (Invitrogen) following the manufacturer’s protocol and then incubated for 72 h. After that, the cells were washed, infected with renilla reporter virus (JcR-2a, kindly provided by R. Bartenschlager [48] for 4 h, and washed again before medium change. At 48 h post infection, the cell supernatants were collected and the cells were lysed and analysed with western blot assay using anti-hCD59 (SC-133171) for KD efficiency and RNA replication using luciferase assay. The collected supernatant were subjected to virus lysis assay as described above and then re-infected to fresh Huh 7.5 cells. At 48 h post re-infection, the cells were lysed and analysed with renilla luciferase assay for virus infectivity level.

### Flow Cytometric Analysis of Complement Regulators/receptors on Infected and Non-infected Huh 7.5 Cells

Huh 7.5 (3×10^5^) cells were washed twice with PBS and incubated with 1 µg anti- CD59, anti-CD55, anti-CD46 or isotope control Abs for 30 min at 4°C. Cells were washed and further incubated with anti- mouse FITC labeled antibody for 20 min at 4°C. Samples were washed again and analysed on a FACS Canto II (Becton Dickinson). To test the expression of CD59 in HVC-infected cells, Huh 7.5 cells (8×105) were seeded in a 6-well plate, infected with Jc1 overnight or mock treated, and incubated for 72 h. Then, cells were washed, harvested, and fixed in 3% PFA before 1 h incubation with anti-hCD59 (MEM-43 by Abcam, 1∶100 in 3% BSA-PBS) at RT. After that, the cells were washed with PBS and incubated with secondary antibody (Alexa Fluor 488; Invitrogen, 1/500 in PBS) for another 1 h in RT.

### Complement-mediated Cytotoxicity Assay

NHS (25%) or h.i.NHS (25%) were incubated together with anti-env and/or RCA antibodies (15 µg/ml) and UV-inactivated HCV Jc1 for 1 hour at 37°C. Then, 100 µl of the mixture was applied to Huh 7.5 cells (2×10^5^) and further incubated for 1 hr at 37°C. Propidium iodide was added to the reaction mixture for the last 5 minutes and measurement was carried by flow cytometric analysis of propidium iodide (PI)-negative and positive by counting the living cells for 60 s on a FACS Canto II as described elsewhere [49], [50], [51].

### Statistical Analysis

Statistical analyses were performed with GraphPad Prism software using the unpaired students t test or one way ANOVA as indicated in the figure legends. Values below 0.05 were considered as significant.

## Supporting Information

Figure S1 **Effect of hCD59 siRNA KD on hCD59 expression.**
(PDF)

Figure S2 **Cytotoxicity assay using Huh7.5 cells.**
(PDF)
