# Supplementary material for: Specific Acquisition of Functional CD59 but Not CD46 or CD55 by Hepatitis C Virus
Source: PLoS One. 2012 Sep 25;7(9):e45770. doi: 10.1371/journal.pone.0045770 (PMC3458075; doi:10.1371/journal.pone.0045770)
Supplement: Figure S1 — Effect of hCD59 siRNA KD on hCD59 expression. (PDF) [file pone.0045770.s001.pdf]

Figure S1: Effect of hCD59 siRNA KD on hCD59 expression

PFA 3%
+ Saponin 0,5%
1st Ab 1:200 ON - 4C
2nd Ab 1:1000 alexa 488
2,5h - RT

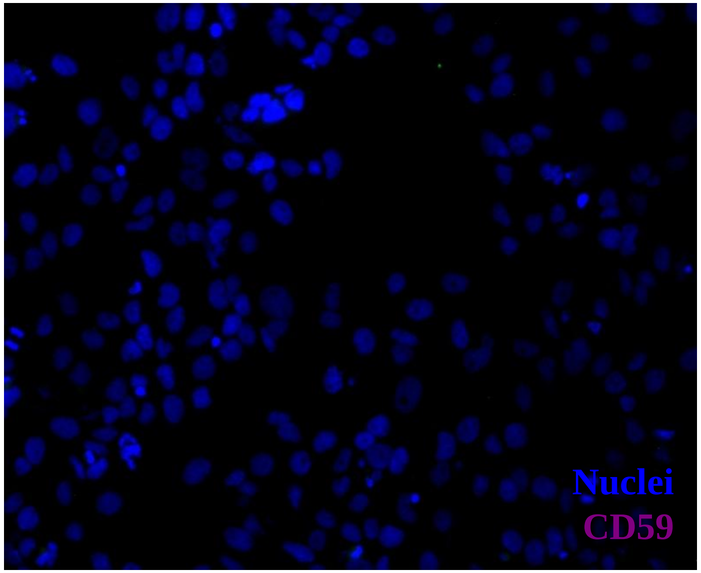

NO 1st Ab

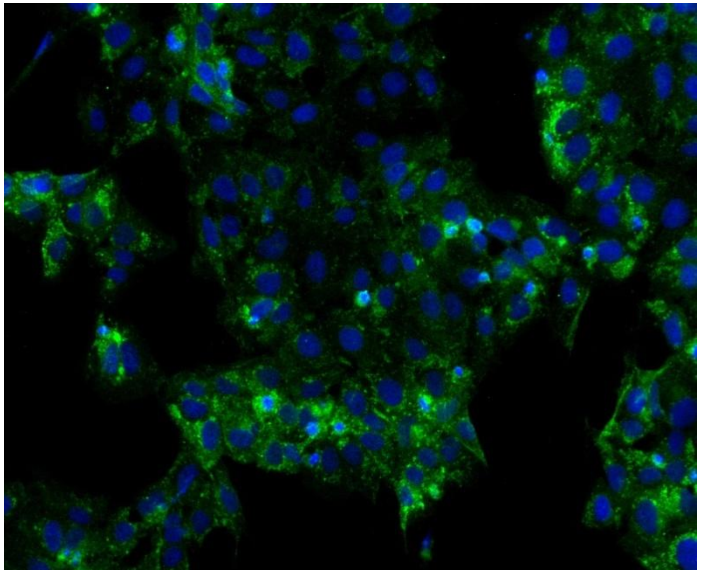

Non targeting

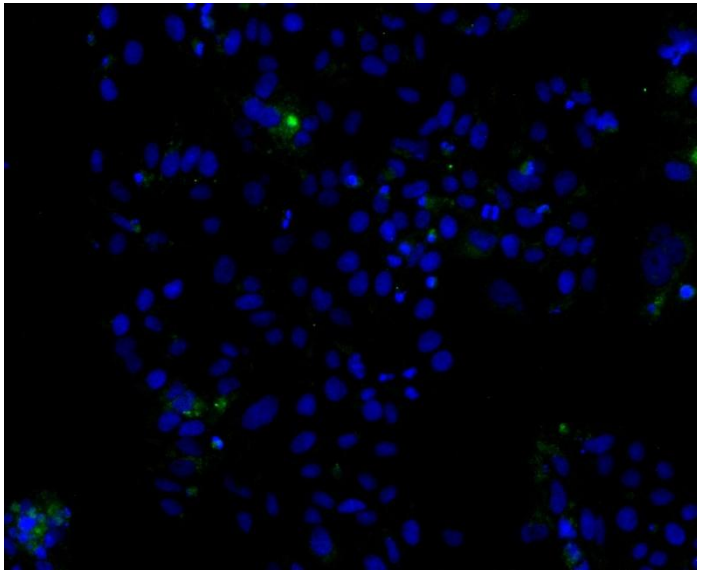

siCD59 – 25nM

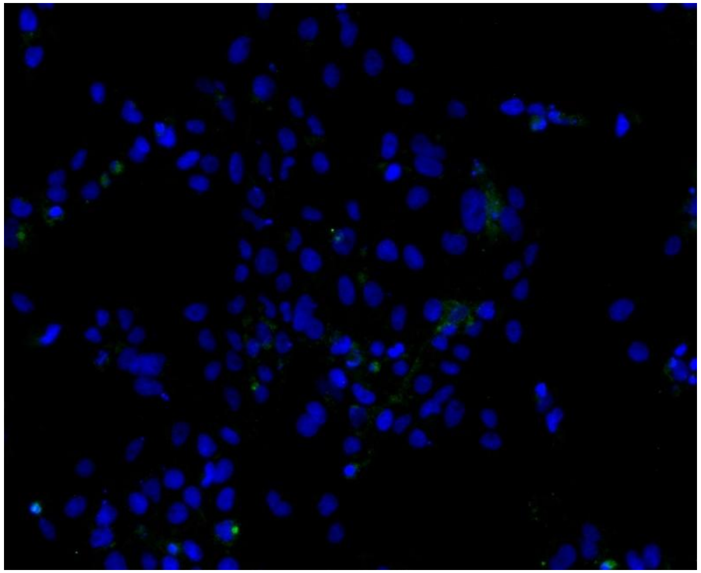

siCD59 – 50nM

3d KD
