# Supplementary material for: Specific Acquisition of Functional CD59 but Not CD46 or CD55 by Hepatitis C Virus
Source: PLoS One. 2012 Sep 25;7(9):e45770. doi: 10.1371/journal.pone.0045770 (PMC3458075; doi:10.1371/journal.pone.0045770)

Figure S2. Cytotoxicity assay using Huh7.5 cells. UV-inactivated cell free HCV(Luc-JC1) was incubated with a combnation of anti-env either with anti-CD59 or anti-CD55 in the presence of 25%NHS or h.i.NHS. The mixture was applied to further incubation with Huh 7.5 cells. Cytolysis was monitored by counting propidiumiodide negative cells by FACS.

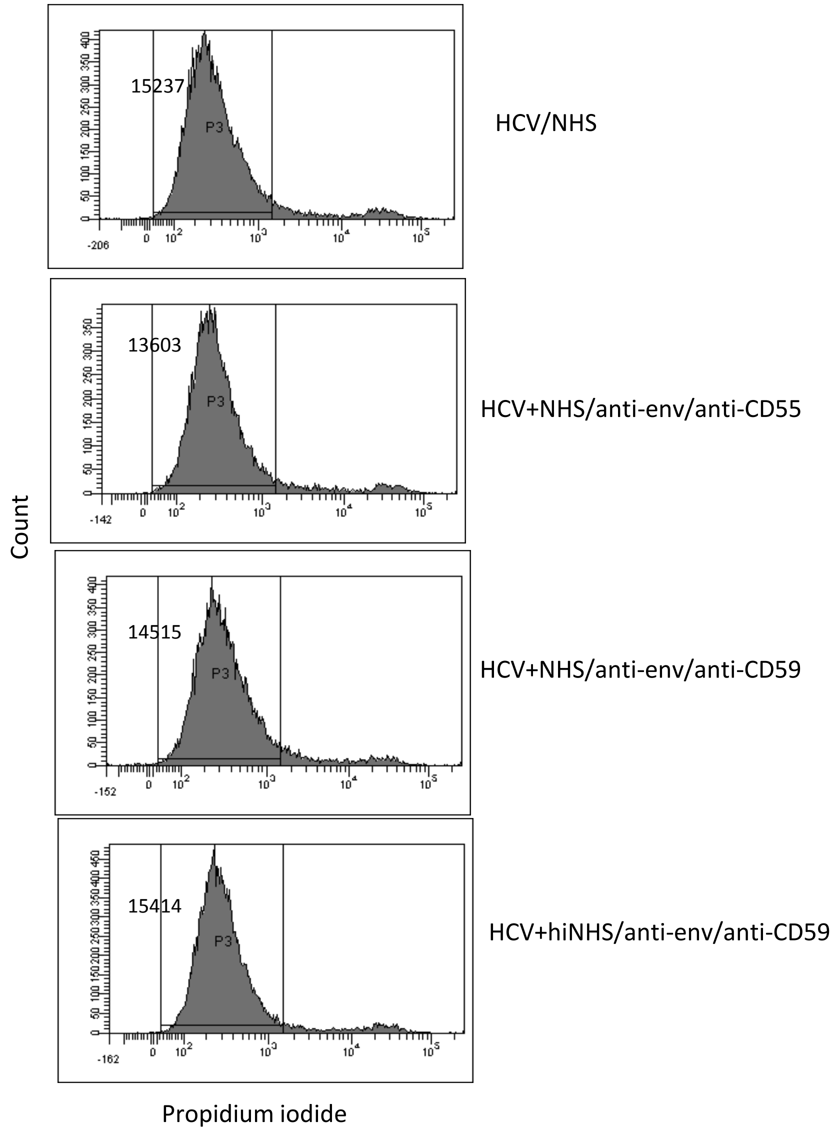

Supplement: Figure S2 — Cytotoxicity assay using Huh7.5 cells. (PDF) [file pone.0045770.s002.pdf]
